# High-throughput sequencing unravels placental vascular dysfunction and oxidative stress as mechanistic drivers of advanced maternal age-associated pregnancy

**DOI:** 10.3389/fgene.2025.1636834

**Published:** 2025-08-13

**Authors:** Xin Sun, Yao Chen, Feifei Hu, Yumeng Qiao, He Wu, Yao Su, Yue Hu, Jie Wu, Mingli Huang

**Affiliations:** ^1^ Obstetrical Department, The First Affiliated Hospital of Harbin Medical University, Harbin, Heilongjiang, China; ^2^ Pathology Department, The First Affiliated Hospital of Harbin Medical University, Harbin, Heilongjiang, China; ^3^ Key Laboratory of Preservation of Human Genetic Resources and Disease Control in China (Harbin Medical University), Ministry of Education, Harbin, Heilongjiang, China; ^4^ Department of Medical Genetics, School of Basic Medical Sciences, Harbin Medical University, Harbin, Heilongjiang, China

**Keywords:** placental transcriptome sequencing, placental vascular injury, oxidative stress, advanced maternal age, SIRT3

## Abstract

**Introduction:**

Women with advanced maternal age (AMA) face a higher risk of pregnancy complications including preeclampsia, fetal growth restriction, and preterm birth. While placental dysfunction is implicated, the underlying mechanisms remain unclear. This study employs high-throughput sequencing-based transcriptomics to investigate AMA-associated dysregulation in placental angiogenesis, exploring links to redox imbalance. Our goal is to establish mechanistic and functional links between altered gene expression and perinatal complications.

**Methods:**

Placental pathology from 129 cases was analyzed to identify risk factors for maternal vascular malperfusion (MVM), a key pathological condition known to impair placental function. Building upon this pathological context, placental RNA-seq data from AMA and control pregnancies, combined with public datasets, were analyzed to identify differentially expressed genes (DEGs). Gene Ontology (GO) and Kyoto Encyclopedia of Genes and Genomes (KEGG) enrichment analyses were performed to identify functional pathways of dysregulated genes. Weighted gene co-expression network analysis (WGCNA) was utilized to detect AMA-related modules and hub genes, which were subsequently validated via Western blotting, qPCR, and immunohistochemistry (IHC).

**Results:**

Analysis of placental pathology (n = 129) identified advanced maternal age (AMA) as an independent risk factor for maternal vascular malperfusion (MVM) (OR = 3.022, 95% CI 1.337–6.832). RNA-seq revealed 731 differentially expressed genes (DEGs) in AMA placentas, which were enriched in energy metabolism, oxidative stress, angiogenesis, and NAD(P)H metabolic pathways. Weighted gene co-expression network analysis (WGCNA) identified six co-expression modules, of which the black module (most strongly AMA-associated) contained six hub genes (SIRT3, TLR6, AOX1, ARG1, CRYAB, HGF) exhibiting high intramodular connectivity. Functional studies confirmed that placental SIRT3 expression was markedly reduced in AMA (P < 0.05), while both impaired vascular perfusion and oxidative stress were significantly more severe.

**Conclusion:**

Our findings indicate that reduced placental SIRT3 expression is a key molecular feature in advanced maternal age. This reduction may be related to increased risk of maternal vascular malperfusion and adverse pregnancy outcomes, potentially through mechanisms involving exacerbated oxidative stress and impaired placental vascular function; however, further studies are needed to clarify these associations.

## 1 Introduction

In recent decades, a marked increase in pregnancies among women of advanced maternal age (AMA) has been observed across both developed and developing nations. Generally, AMA is designates as age ≥35 years at delivery, and very advanced maternal age typically refers to those over 40 or 45 years (Dong et al., 2017). The rising prevalence of pregnancies among women of AMA stems from multiple socioeconomic and medical factors, including the widespread adoption of family planning methods, career-oriented delays in childbearing, and significant advancements in assisted reproductive technologies. Consequently, there has been growing concern regarding the potential negative impacts of advanced maternal age on perinatal outcomes. Accumulating epidemiological evidence demonstrated that AMA significantly elevates the risks of intrauterine growth restriction, preterm birth, low birth weight infants, miscarriage, and placenta previa (Kahveci et al., 2018; Rodrigues et al., 2023; Tembo et al., 2020). Furthermore, women of AMA exhibit significantly higher risk to common pregnancy-related complications such as gestational hypertension and gestational diabetes mellitus. These findings highlight the elevated health risks accompanying pregnancies in older women, necessitating rigorous prenatal monitoring and management. The mechanisms underlying the increased incidence of complications and adverse pregnancy outcomes in AMA remain poorly understood, and current clinical practice lacks established interventions specifically designed to address obstetric risks in AMA pregnancies. Therefore, elucidating the molecular mechanisms underlying heightened obstetric complications in this population is essential for improving maternal-fetal outcomes in AMA pregnancies.

The human placenta, a transient yet critical interface between maternal and fetal circulations, requires precisely regulated vascular perfusion to maintain its physiological functions. Murine studies modeling AMA pregnancies have demonstrated significant placental developmental defects, which likely constitute a pathophysiological basis for the elevated incidence of gestational disorders associated with AMA (Woods et al., 2017). The 2016 Amsterdam Workshop established a unified classification and definitions for various pathological changes in the placenta, introducing the concept of maternal vascular malperfusion (MVM) (Khong et al., 2016). MVM characterized by histopathological features including placental parenchyma and maternal vascular abnormalities, primarily attributed to impaired remodeling of the spiral arteries, leading to inadequate placental bed circulation. The histopathological lesions associated with MVM primarily include decidual vascular disease, placental villous dysplasia, and accelerated villous maturation, all of which could drive placental underdevelopment (Zhang D. C. et al., 2020). Accumulating evidence has consistently established that MVM is associated with an increased incidence of adverse pregnancy outcomes, including preeclampsia, eclampsia, HELLP syndrome, spontaneous preterm birth, fetal growth restriction, reduced fetal movements at term, and even intrauterine fetal demise (Burton and Jauniaux, 2018; Bustamante Helfrich et al., 2017; Kulkarni et al., 2021; Melamed et al., 2021). Miremerg et al. reported a higher incidence of MVM in placentas from older pregnancies, marking the first evidence of an association between AMA and MVM (Miremerg et al., 2020). Furthermore, MVM may result in angiogenic imbalance, systemic vasoconstriction, and the production of circulating placental-derived microvesicles. These pathological mediators collectively induce oxidative stress, driving damage to the maternal vasculature, including the placenta, which may persist postpartum (Catov et al., 2022). The oxidative stress-mediated injury to placental endothelial cells has been closely linked to various pregnancy-related complications including preeclampsia, fetal growth restriction, preterm birth and recurrent miscarriage (Sultana et al., 2017). Studies demonstrate that AMA mice models display heightened oxidative stress within uterine and placental compartments, with antioxidant supplementation improving placental functional and morphological placental integrity (Silva et al., 2015). In contrast, the age-related impacts on oxidative stress pathways in human placental systems remain poorly characterized.

The effects of aging on pregnancy are complex and intricate, with mammalian fertility experiencing a rapid decline as maternal age increase. This decline is largely attributed to an exponential rise in chromosomal segregation errors in oocytes with advancing age. AMA is associated with a higher incidence of pregnancy complications and birth defects, many of which occur even in cases with normal embryonic karyotypes, including preeclampsia, fetal growth restriction, preterm birth, and late miscarriage (Pinheiro et al., 2019; The impact of advanced maternal age on pregnancy outcome, 2021). However, current research on age-related alterations in placental gene expression remains remain limited. Notably, Kokorudz et al. found significant downregulation of several trophoblast-related genes in the placentas of AMA mice, correlating with placental dysfunction and adverse maternal-fetal outcomes (Kokorudz et al., 2022). Additionally, placentas from older pregnancies exhibit signs of aging, characterized by increased expression of p53, p16, and p21, as well as a marked rise in senescence-associated β-galactosidase (Chen et al., 2021). Systematic investigation of age-dependent transcriptional variations in placental tissue may provide insights into the underlying mechanisms by which AMA heightens pregnancy risks.

In the present study, we conducted high-throughput transcriptome sequencing on placental tissues from pregnancies of varying reproductive ages and identified DEGs associated with AMA. WGCNA analysis was performed to identify candidate hub genes related to AMA, and further molecular analysis of clinical placental tissues from AMA pregnancies revealed downregulation of SIRT3, which correlated with impaired placental vascular perfusion and increased oxidative stress.

## 2 Materials and methods

### 2.1 Placental pathology results collection

Retrospective analysis of placental pathology results from pregnant women who received regular prenatal care and delivered at The First Affiliated Hospital of Harbin Medical University between September 2021 and February 2024. Based on placental pathology, 129 cases were classified into the MVM group with 57 cases and the non-MVM group with 72 cases. Maternal characteristics (age, gravidity, BMI, gestational age) and pregnancy-related complications and comorbidities (hypertensive disorders, thyroid dysfunction, gestational diabetes, placental implantation diseases) were analyzed. All placental samples were obtained with informed consent, and the study was approved by the local Ethics Committee (Ethics Approval No. 2024381), in compliance with the Declaration of Helsinki.

This study defined the following inclusion criteria for placental pathology results collection: 1) Placenta gross appearance without significant abnormalities; 2) Complete prenatal and delivery records of the mother; 3) Informed consent was obtained from both the mother and her family.

The exclusion criteria for placental pathology results collection were as follow: 1) presence of severe liver dysfunction, renal impairment, hematological diseases, congenital genetic disorders, or autoimmune disorders; 2) fetal developmental abnormalities; 3) multiple pregnancies, stillbirths, or fetal demise; 4) abnormal placental appearance; 5) incomplete clinical data.

### 2.2 Collection and processing of placental and peripheral blood samples

Placental tissue from the central region was collected during cesarean delivery under sterile conditions. Each sample weighed approximately 100 mg and was rinsed with non-enzymatic physiological saline (Solarbio) to remove any residual blood. The tissue was then blotted dry and immersed in a preservation solution (Beyotime) at a ratio of 1:10 (tissue to solution). The samples were stored overnight at −4 °C and subsequently transferred to a −80 °C freezer for long-term preservation. Additionally, a separate portion of the placental tissue was fixed in 4% paraformaldehyde solution for 48 h, followed by routine paraffin embedding for subsequent immunohistochemical experiments.

Peripheral blood samples (3 mL) were collected from fasting mothers prior to the cesarean delivery. The blood was preserved in collection tubes containing separation gel and clot activator, then refrigerated at 4 °C overnight. The samples were centrifuged at 1,000 *g* for 15 min at 4 °C. The supernatant was collected, aliquoted, and stored at −80 °C for analysis to be conducted within 1 month.

The inclusion criteria for clinical samples were as follows: 1) gestational age of ≥34 weeks; 2) singleton live births; 3) absence of fetal developmental abnormalities; 4) healthy mothers without gestational comorbidities or complications. Participants were grouped by maternal age: 1) the advanced maternal age (AMA) group, comprising women aged between 35 and 50 years; 2) the normal control (NC) group, comprising women aged between 20 and 34 years. The exclusion criteria were as same as those in placental pathology results collection.

### 2.3 ELISA

Prior to the experiment, samples were retrieved from the −80 °C freezer. A total of 25 μL of each sample was mixed with 225 μL of diluent in sterile EP tubes, resulting in a 10-fold dilution. After incubating at room temperature for 1h, required assay plates were removed from their aluminum foil packaging. Any remaining plates were resealed in self-sealing bags and stored at 4 °C. Standard, blank, sample, and control wells for the sample diluent were set up, with each condition prepared in triplicate. Following the procedure outlined in the ELISA kit manual (Mlbio), samples were added in the designated order. After adding the stop solution, optical density (OD) values were measured within 15 min at 450 nm using a microplate reader.

### 2.4 Assessment of placental oxidative stress level

To assess oxidative stress levels in placental tissue, 100 mg of placental tissue was minced and then mixed with physiological saline at a volume ratio of 1:9. The tissue was then homogenized using a tissue grinder. The mixture was centrifuged at 3,000 rpm for 5 min to obtain the supernatant, which served as a 10% tissue homogenate. Protein concentration in the homogenate was quantified using the BCA Protein Assay Kit (Beyotime), following the manufacturer’s instructions. For malondialdehyde (MDA) quantification, samples were processed according to the MDA Assay Kit protocol (NJJCBIO). The samples were incubated in a boiling water for 40 min, then cooled to room temperature under flowing water and centrifuged at 100 rpm for 10 min. The supernatant was collected, and absorbance was measured at 530–540 nm using a microplate reader to calculate the MDA content. In addition, another aliquot of the 10% placental tissue homogenate was processed using the Superoxide Dismutase (SOD) Assay Kit (NJJCBIO). Samples were incubated at 37 °C for 40 min, followed by the addition of a color reagent, and absorbance was measured at 550 nm.

### 2.5 Immunohistochemistry

Tissue sections (4 μm) were prepared using an automatic microtome and placed in an incubator for 30 min to facilitate dehydration and rehydration. Antigen retrieval was performed via microwave for 10 min, followed by cooling of the slides to room temperature and washing three times with PBST. Nonspecific binding sites of sections were blocked with 10% goat serum (1 h) and incubated with the primary antibody overnight at 4 °C. After PBST washes, the secondary antibody was applied (1 h), followed by DAB staining for 5 min, and hematoxylin counterstaining for 30 s. Slides were examined using an optical microscope (×200), and SIRT3-positive cells in interstitial regions were counted in each field. Immunohistochemical (IHC) staining was evaluated by two pathologists using a scoring system: no staining (“none”) was assigned a score of 0 for both intensity and distribution; weak staining (“weak”) received a score of 1 for intensity and a distribution of 1%–25%; moderate staining (“moderate”) received a score of 2 for intensity and a distribution of 26%–50%; strong staining (“strong”) received a score of 3 for intensity and a distribution of 51%–75%; and very strong staining (“very strong”) was assigned a score of 4 for intensity with a distribution of 75%–100%. The cumulative score was calculated as the product of staining intensity and distribution.

### 2.6 Western blot

Placental tissue (100 mg) was ground in liquid nitrogen, lysed in 1 mL RIPA buffer containing PMSF, and sonicated for 1 min. After incubation on ice for 30 min with mixing, the lysate was centrifuged (12,000 rpm, 25 min, 4 °C), and the supernatant was collected for protein quantification using a BCA protein assay kit. Proteins were mixed with a 5× loading buffer (4:1), denatured at 100 °C, aliquoted (200 μL), and stored at −20 °C. For SDS-PAGE, 8 μL of protein sample and 3 μL of molecular weight marker were loaded per lane and separated at 200 V for 25 min). Proteins were transferred onto a PVDF membrane (300 mA, 25 min, ice-cooled). The membrane was blocked (5% BSA, 1 h), incubated overnight at 4 °C with primary antibodies (anti-SIRT3 and anti-β-actin), and subsequently secondary antibodies at room temperature for 1 h. After washing with TBST, protein bands were visualized using enhanced chemiluminescence (ECL) and imaged on a Tanon-520 Gel Imaging System.

### 2.7 qRT-PCR

The primer sequences for human SIRT3 were as follows:

Forward (F): 5′-GAA​GTG​GAG​GCA​GCA​GTG​ACA​AG-3′,
Reverse (R): 5′-CGGC GAT​CTG​AAG​TCT​GGA​ATG​C-3′.

Gene expression in placental tissues was assessed using qRT-PCR, with synthesized cDNA serving as a template. The reaction was conducted using gene-specific primers and a SYBR^®^ Green PCR kit (EZBioscience) on a StepOnePlus Real-Time PCR System (Bio-Rad Biosystems). The thermal cycling conditions included an initial denaturation at 95 °C for 15 min, followed by 40 amplification cycles of 94 °C for 15 s, 60 °C for 30 s, and 72 °C for 30 s. GAPDH was employed as an internal control for normalization. Relative mRNA expression levels were then calculated using the 2^−ΔΔCT^ method.

### 2.8 Bioinformatics analysis of RNA-seq and data source

For the sequencing analysis, a total of 6 samples were randomly chosen from each group. Ensuring each sample integrity throughout the processing. RNA-seq libraries were prepared using the Illumina TruSeq™ RNA Sample Prep Kit, and sequenced on the Illumina Novaseq 6000 platform for high-throughput analysis. Gene expression levels in placental tissues were quantified using FPKM. Principal Component Analysis (PCA) was performed using the FactoMineR v2.6 package in R to assess sample clustering and identify potential outliers. Differential gene expression analysis between the AMA and NC samples was carried out using the DESeq2 v1.34 package. DEGs were defined as those with an adjusted P-value <0.05 and an absolute Fold Change value ≥2. To elucidate the biological function of DEGs, GO and KEGG pathway enrichment analyses were performed utilizing the clusterProfiler v4.2.0 package. Gene Set Enrichment Analysis (GSEA) was utilized to evaluate enrichment results with a normalized enrichment score (|NES|) > 1, a false discovery rate (FDR) q-value <25%, and an adjusted P-value <0.05, indicating statistical significance. Bayesian network analysis of enriched pathways was conducted using CBNplot v1.2.1 to infer regulatory interactions.

The WGCNA package was employed to contruct a gene co-expression network. Initially, sample clustering was performed based on clinical data, with an outlier threshold of 60 applied to exclude aberrant samples. The Pearson correlation matrix was then transformed into a weighted adjacency matrix through the power function f (X) = X^β^, where the optimal soft thresholding power (β) was identified. A power of 5 was selected, and the adjacency matrix was converted into a topological overlap matrix (TOM). The TOM facilitated clustering based on topological overlap (TO) dissimilarity (1 - TOM). Genes were clustered based on TOM using average linkage hierarchical clustering. Gene modules were defined by grouping genes with similar expression patterns, enforcing a minimum module size of 30. Modules with a dissimilarity threshold below 0.25 were merged. Key hub genes within co-expressed modules were identified based on gene significance (GS > 0.2, P-value <0.05) and module membership (MM > 0.8, P-value <0.05). Modules exhibiting the highest correlation with gestational age were selected for further analysis. The genes within these significant modules were intersected with DEGs to filter overlapping candidates. GO and KEGG pathway enrichment analyses of key genes were conducted using the clusterProfiler package. Statistical significance was identified as an adjusted P-value <0.05. The filtered key genes were further analyzed using the STRING database for protein-protein interaction (PPI) network construction. Cytoscape v3.10.1 was used to compute topological parameters of the network. The cytoHubba plugin was utilized to identify core genes associated with gestational age, refining the selection of crucial nodes within the PPI network.

RNA-seq data (GSE133592) from trophoblast cells and clinical records of 32 pregnant women were obtained from the GEO database (https://www.ncbi.nlm.nih.gov/geo), categorized into young (10 cases), intermediate (16 cases), and AMA (6 cases) groups.

### 2.9 Statistical analysis

Data analysis was conducted using SPSS (IBM SPSS Statistics 26.0) and R (version 4.3.0) software. Continuous variables were presented as mean ± standard deviation (SD) or median with interquartile ranges (IQRs), depending on their distribution. Categorical variables were expressed as counts and percentages. For group comparisons, one-way analysis of variance (ANOVA) was applied for normally distributed variables with homogeneous variance. For continuous variables, Student’s t*-*test or the Mann–Whitney *U* test was applied, depending on the data distribution. Logistic regression analysis was employed for the statistical examination of outcomes related to factors and categorical variables. The Fisher exact test or chi-square test was used to compare categorical variables between groups, as appropriate. Statistical graphs were generated using Graphpad 9.5 and R 4.3.0 software.

## 3 Results

### 3.1 AMA as the independent risk factor for MVM

The clinical characteristics of the study cohort are presented in Table 1. Comparing with the Non-MVM group, there were significant increases in age (*p* < 0.05), the proportion of AMA (*p* < 0.05), body mass index (BMI) (*p* < 0.05), and the incidence of pregnancy-induced hypertension (*p* < 0.05) in the MVM group. No statistically significant differences were observed in other indicators, including gestational diabetes, thyroid dysfunction, and placental implantation diseases. Logistic regression analysis was performed to assess the correlation between AMA, BMI, pregnancy-induced hypertension, and MVM, with detailed results in Table 2. The analysis identified AMA as an independent risk factor for MVM (OR = 3.022, 95% CI 1.337–6.832). Additionally, Elevated BMI (OR = 1.145, 95% CI 1.037–1.246) and pregnancy-induced hypertension (OR = 2.671, 95% CI 1.065–6.701) were identified strong independent associations with MVM. Based on retrospective analysis of placental pathology results, AMA was identified as an independent risk factor for maternal vascular malperfusion (MVM). To further investigate the underlying molecular mechanisms, this study performed transcriptome sequencing (RNA-seq) on placental tissues from AMA group (≥35 years) and normal control group (NC group, 20–34 years), aiming to delineate differential gene expression profiles between the cohorts.

**TABLE 1:** Comparison of clinical characteristics and perinatal complications between MVM and Non-MVM groups.

Characteristics	MVM(n = 57)	Non-MVM(n = 72)	P value
Age	33.70 ± 4.66	31.89 ± 3.90	0.018
Advanced maternal age	27 (47.37%)	17 (23.61%)	0.005
Primipara	41 (71.93%)	57 (79.17%)	0.339
BMI(kg/m^2^)	30.12 ± 4.61	27.46 ± 3.87	0.001
Abnormal thyroid function	9 (15.79%)	17 (26.61%)	0.271
Hypertensive disorders	23 (40.35%)	10 (13.89%)	0.001
Gestational diabetes mellitus	22 (38.60%)	19 (26.39%)	0.139
Placenta accreta	24 (42.11%)	23 (31.94%)	0.234
Umbilical cord twisting	19 (33.33%)	26 (36.11%)	0.742

**TABLE 2:** Multivariate logistic regression analysis of perinatal factors affecting MVM.

Variable	OR	95%CI	P value
Advanced maternal age	3.022	1.337–6.832	0.008
BMI	1.145	1.037–1.246	0.007
Hypertensive disorders	2.671	1.065–6.701	0.036

### 3.2 DEGs in the placenta of pregnant woman with AMA

**FIGURE 1:**
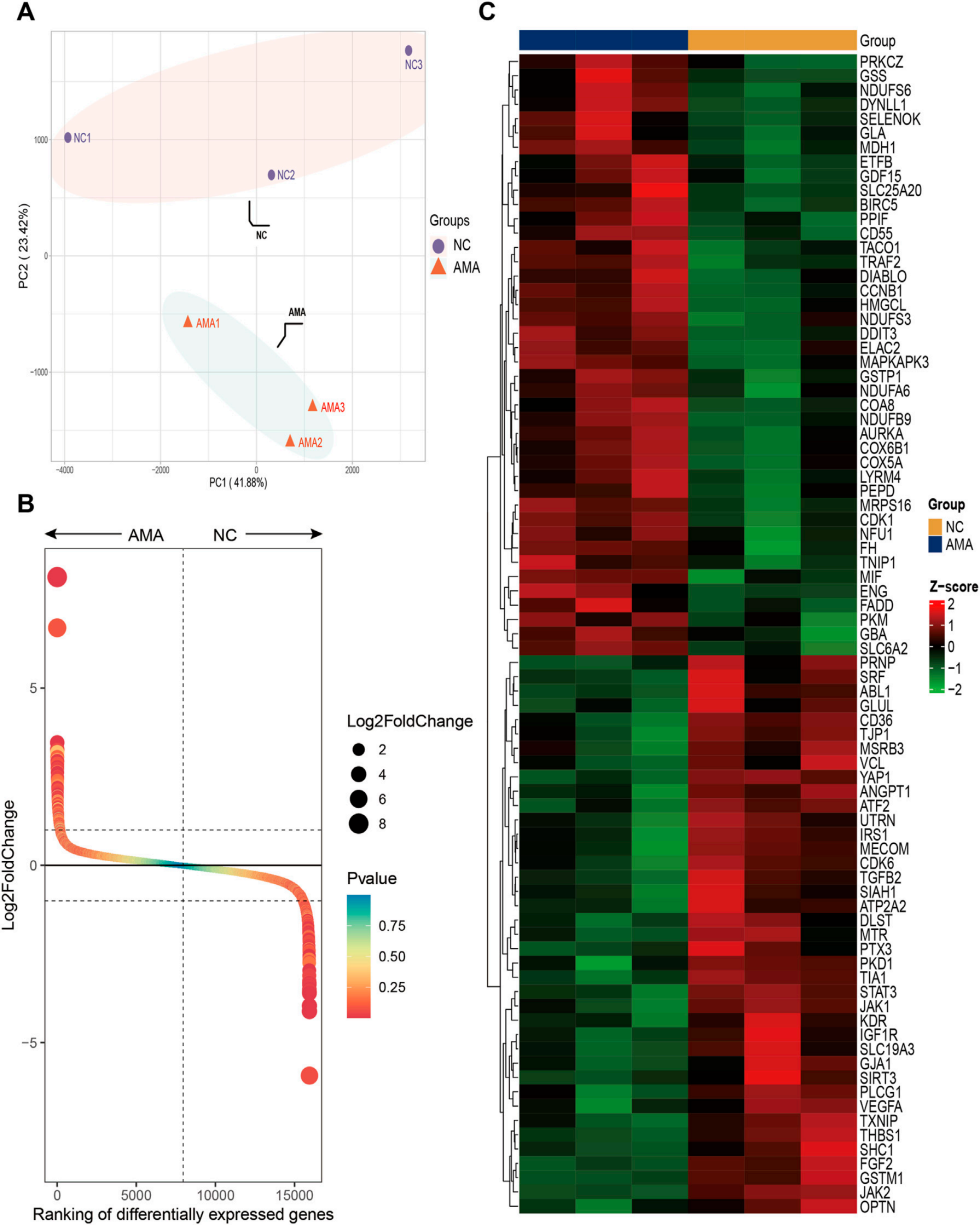
High-Throughput Sequencing Analysis of Gene Expression in Placental Tissues from AMA and NC Groups **(A)** The PCA score plot illustrates the transcriptional differences between the two groups, highlighting the distinct expression profiles of the placental tissues from AMA and NC pregnancies. **(B)** The Gene rank plot presents the top upregulated and downregulated genes between the AMA and NC groups, showcasing significant variances in gene expression levels associated with the two distinct conditions. **(C)** The heatmap displays the top 50 upregulated and downregulated genes, providing a visual representation of the expression patterns, where clustering of genes reveals the relationships and differences in expression levels across the samples from both groups.

Principal component analysis (PCA) demonstrated distinct inter-group separation in transcriptional profiles, whereas samples within each group exhibited cohesive clustering patterns (Figure 1A). A total of 731 significantly differentially expressed genes (DEGs) were identified from the AMA group compared to the NC group (|log2FC| > 1, adjusted *p* < 0.05), with 445 genes upregulation and 286 genes downregulation in the AMA group. DEGs were ranked by log2 (fold change) magnitude (Figure 1B), with the top most significantly 50 upregulated and downregulated genes visualized through heatmap analysis (Figure 1C).

### 3.3 Multiple analysis of DEGs related to AMA in GO, KEGG and GSEA

**FIGURE 2:**
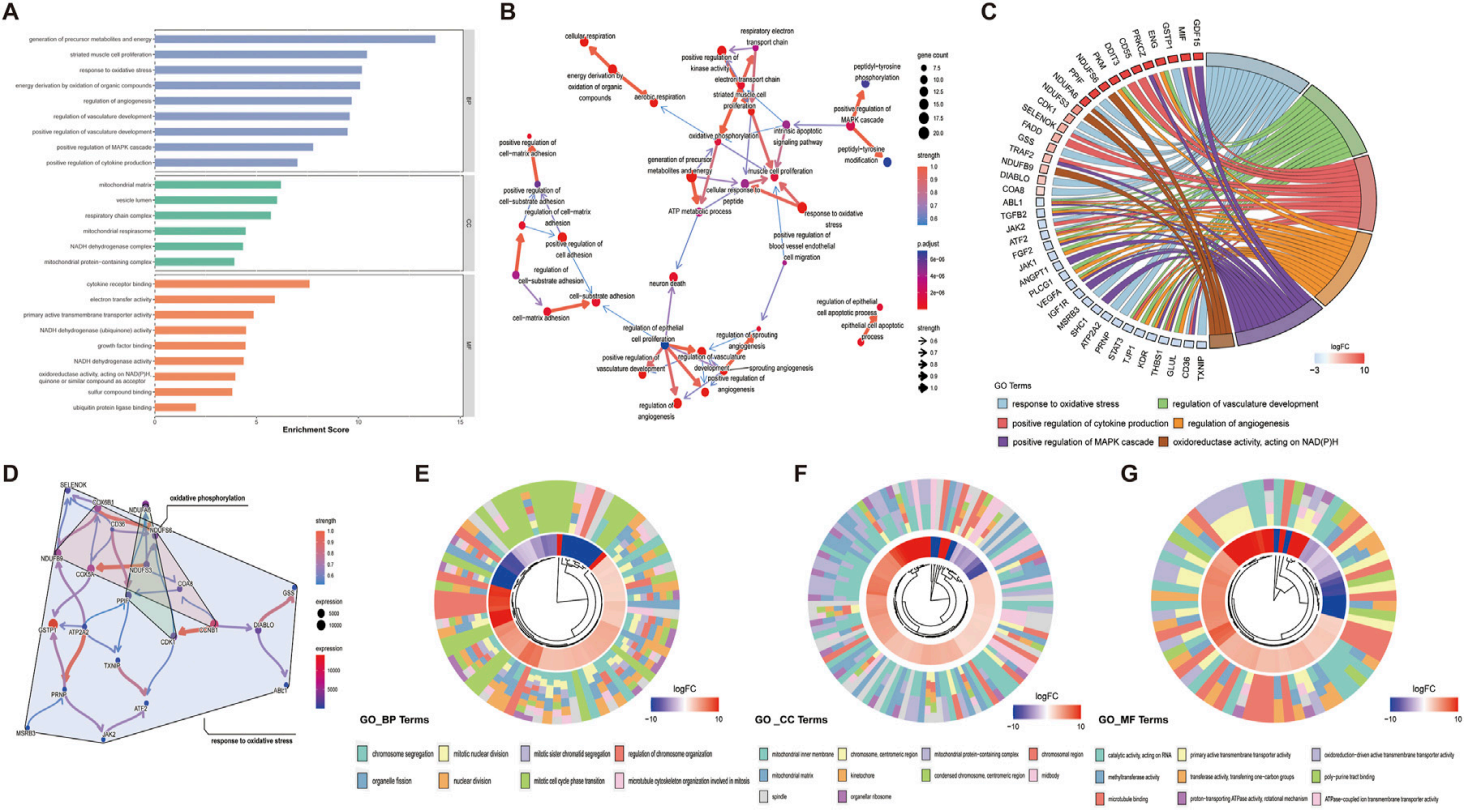
GO Enrichment Analysis of Differential Genes in Placental Tissues from AMA and NC Groups **(A)** The bar chart illustrates the results of the GO functional enrichment analysis for the DEGs, highlighting significant biological processes, cellular components, and molecular functions associated with the identified DEGs. **(B)** The Bayesian network plot demonstrates the regulatory relationships between key GO processes, providing insights into the interactions and hierarchies among the biological functions influenced by the DEGs. **(C)** The chord diagram reveals the connections between core DEGs related to oxidative stress, angiogenesis, MAPK signaling pathways, and cytokine production processes, emphasizing the integrated roles of these genes in the respective pathways. **(D)** The Bayesian network displays the regulatory relationships among the genes within the oxidative stress and angiogenesis pathways, elucidating the network of interactions that contribute to these critical biological processes. **(E**–**G)** Circular plots visualized key GO terms and functional enrichments of DEGs across three major categories.

**FIGURE 3:**
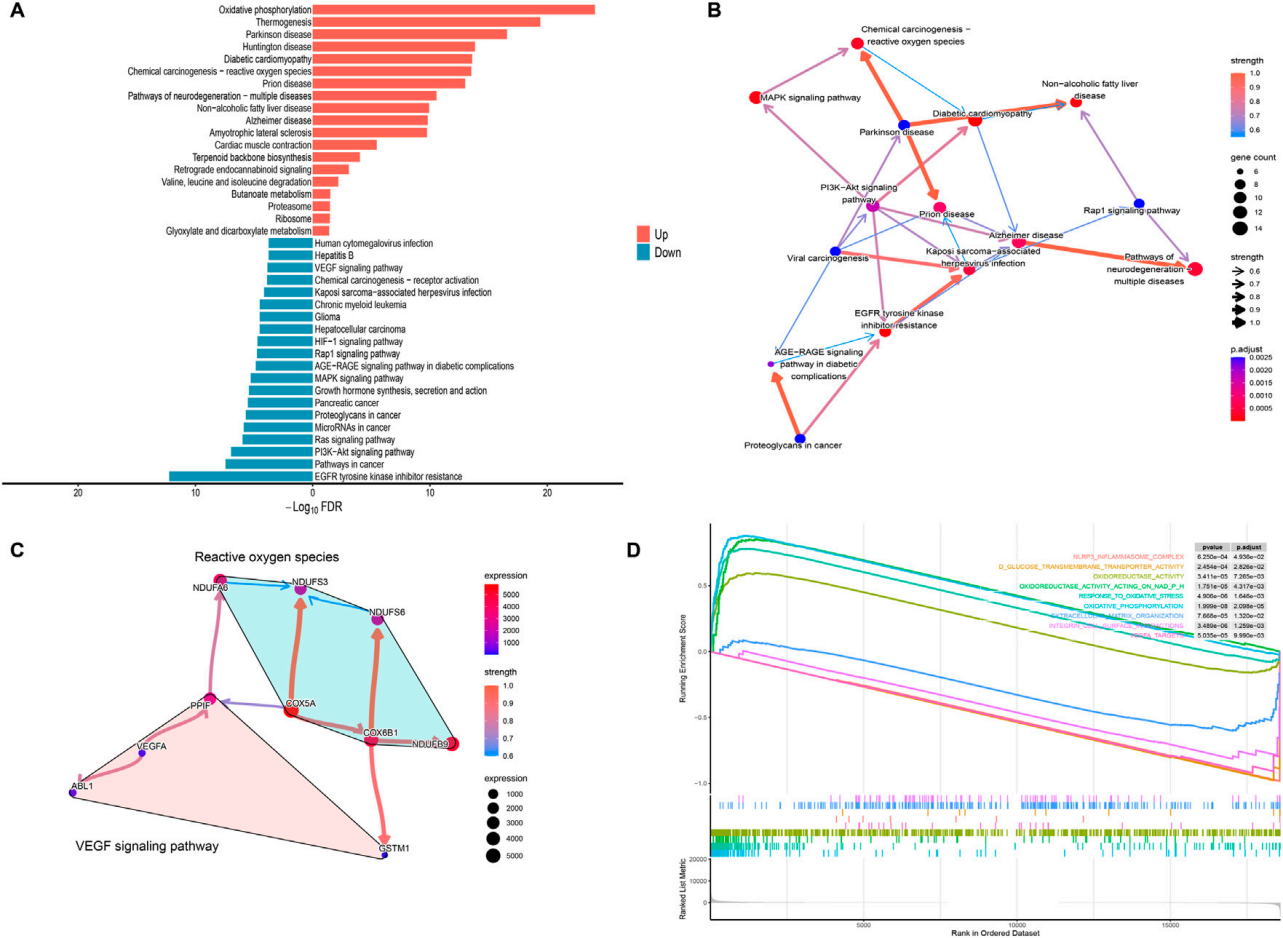
KEGG Enrichment and GSEA Analysis of Differential Genes **(A)** The bidirectional bar chart illustrates the top 20 upregulated and downregulated KEGG pathways, highlighting the significant signaling pathways associated with the DEGs and their roles in the biological context of the study. **(B)** The Bayesian network plot depicts the regulatory relationships among key KEGG processes, further elucidating the interconnectedness of biological functions influenced by the DEGs within the signaling pathways. **(C)** The Bayesian network shows the regulatory relationships among the genes associated with oxidative stress and the VEGF signaling pathway, providing insights into the interactions that may affect angiogenesis and metabolic processes in the placenta. **(D)** The GSEA enrichment score analysis presents representative results, where the enrichment scores for all genes are plotted as a continuous line, with peaks indicating significant enrichment. The gray plot illustrates the rank distribution of all genes, offering a comprehensive view of how gene enrichment correlates with the pathways of interest.

Systematic functional profiling of DEGs was performed through bioinformatics framework, incorporating GO term enrichment, KEGG pathway mapping, and GSEA. GO enrichment analysis of DEGs across different maternal age groups was performed, subsequently identifying the top-ranked entries within the three standard GO categories: biological processes (BP), cellular components (CC), and molecular functions (MF) (Figure 2A). The results from the GO analysis indicated that DEGs are primarily enriched in processes including energy metabolism, oxidative stress, angiogenesis, and NAD(P)H metabolism. Among these processes, the pathways associated with oxidative stress and angiogenesis are identified as critical enrichment pathways, suggesting their involvement in placental development in pregnancies affected by AMA (Figures 2B–D). The circular plot was employed to visually emphasize key entries associated with the three major GO categories, while providing a comprehensive overview of the functional enrichments corresponding to the differentially expressed genes (DEGs) identified in this study (Figures 2E–G). The KEGG pathways analysis categorized biological metabolic pathways into six distinct categories: Metabolism, Genetic Information Processing, Environmental Information Processing, Cellular Processes, Organismal Systems, and Human Diseases. The top 20 representative KEGG enrichment results are illustrated in Figure 3A, with the regulatory relationships among pathways displayed in Figure 3B. The upregulated pathways include oxidative phosphorylation and reactive oxygen species (ROS) production, while the downregulated pathways encompass resistance to EGFR tyrosine kinase inhibitors, PI3K-AKT signaling, and the VEGF signaling pathway. During placental angiogenesis and metabolic processes, ROS signaling pathway and the VEGF signaling pathway have been shown to interact, as illustrated in Figure 3C. Additionally, GSEA demonstrated significant enrichment of DEGs in pathways related to oxidative phosphorylation, oxidative stress, and NAD(P)H metabolism. In contrast, downregulation was predominantly observed in pathways inflammasome formation and responses to VEGFA, as shown in Figure 3D.

### 3.4 Identification of modules related to AMA and screening of hub genes based on WGCNA

**FIGURE 4:**
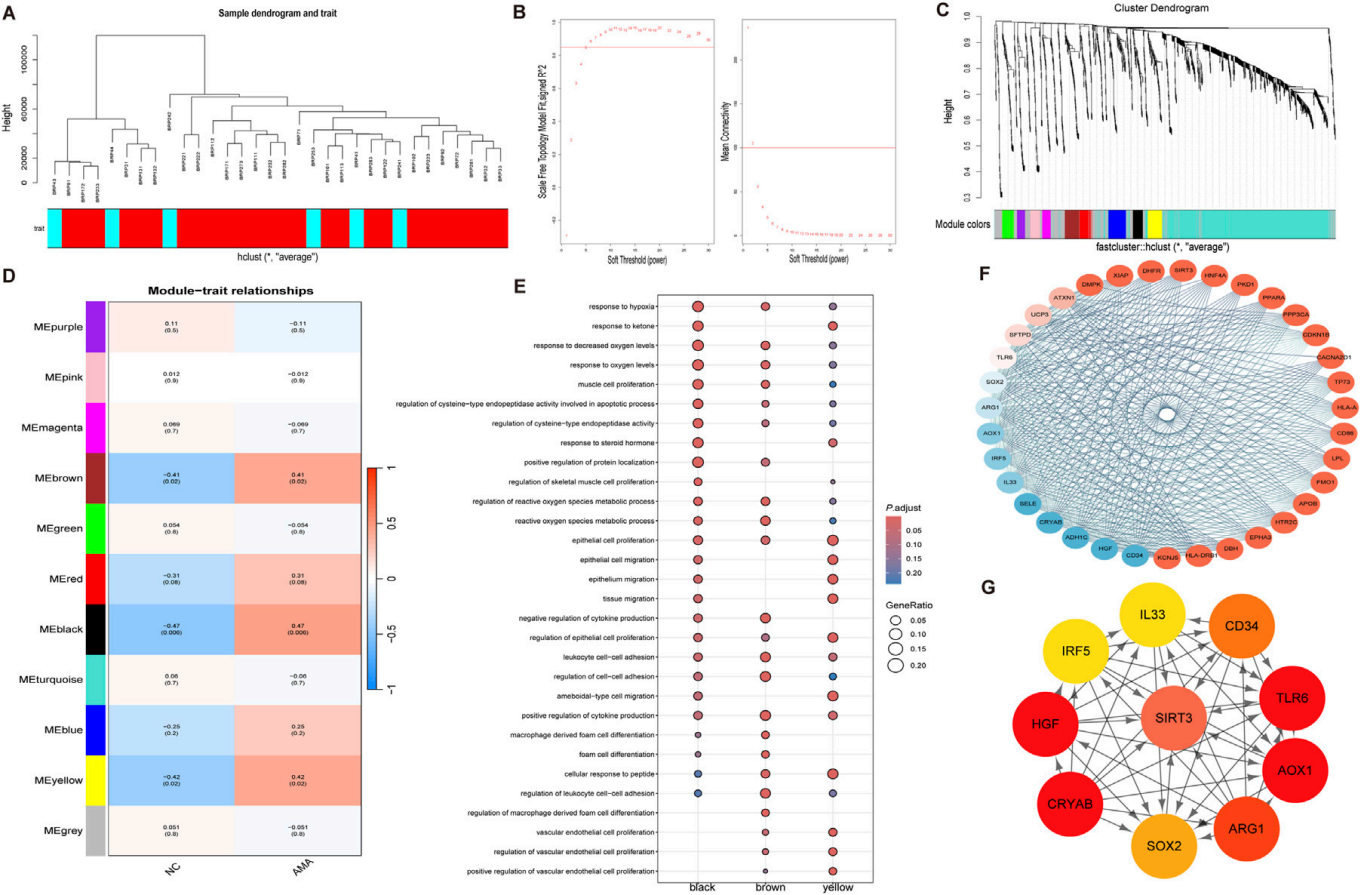
WGCNA Analysis of Core Genes Associated with Maternal Age **(A)** The sample clustering analysis based on weighted correlation illustrates the relationships among samples, allowing for the identification of distinct clusters based on maternal age-related gene expression patterns. **(B)** The determination of the soft threshold parameter in WGCNA is depicted, showcasing the scale-free fit index at various soft threshold parameters (β) and the corresponding average connectivity, which aids in establishing the optimal threshold for network construction. **(C)** The dendrogram generated from clustering all differentially expressed genes based on dissimilarity (1-TOM) illustrates the hierarchical relationships among the genes, providing insights into co-expression patterns within the dataset. **(D)** The heatmap demonstrates the correlations between module eigengenes and various clinical phenotypes associated with maternal age, highlighting the significant relationships and potential clinical relevance of the identified gene modules. **(E)** The dot plot of GO enrichment analysis for the identified characteristic modules presents the enriched biological processes, cellular components, and molecular functions, underscoring the functional relevance of the core genes within the context of maternal age. **(F)** The protein-protein interaction network of key genes within the brown module is displayed, illustrating the interrelations and potential interactions among the genes that may play critical roles in the biological processes studied. **(G)** The key genes from the interaction network identified using cytoHubba are shown, emphasizing the most influential genes within the protein interaction network and their potential significance in maternal age-related pathology.

To identify the most relevant gene modules associated with placental tissue samples of AMA, WGCNA was performed on the GSE133592 dataset for gene clustering (Figure 4A). A co-expression network was constructed based on gene expression profiles. Scale-free genes from this network were obtained using β (soft threshold parameter) = 5, with *R* ^2^ = 0.85 through transformation of the weighted adjacency matrix of the TOM (Figure 4B). Hierarchical clustering dendrogram analysis of the co-expression network revealed 11 distinct co-expressed gene modules, as illustrated by color-coded branches in Figure 4C. The gray module contained unassigned genes, while the black module (51 genes) demonstrated the strongest positive correlation (correlation coefficient = 0.47) with AMA clinical phenotypes (Figure 4D). GO enrichment analysis of the black module revealed significant enrichment of age-associated biological processes, including hypoxia response, oxidative stress response, ketone body metabolism, and endothelial cell proliferation pathways (Figure 4E). PPI network analysis conducted via the STRING database (Figure 4F) identified top hub genes using CytoHubba plugin and Maximum Neighborhood Component (MNC). Ten high-scoring genes were prioritized, with six core hub genes (SIRT3, TLR6, AOX1, ARG1, CRYAB, and HGF) exhibiting the highest connectivity. Among these, SIRT3 demonstrated the maximal degree score, indicating its central regulatory role within the network (Figure 4G).

### 3.5 SIRT3 downregulation in placenta of AMA associated with impaired angiogenesis and oxidative stress imbalance

**FIGURE 5:**
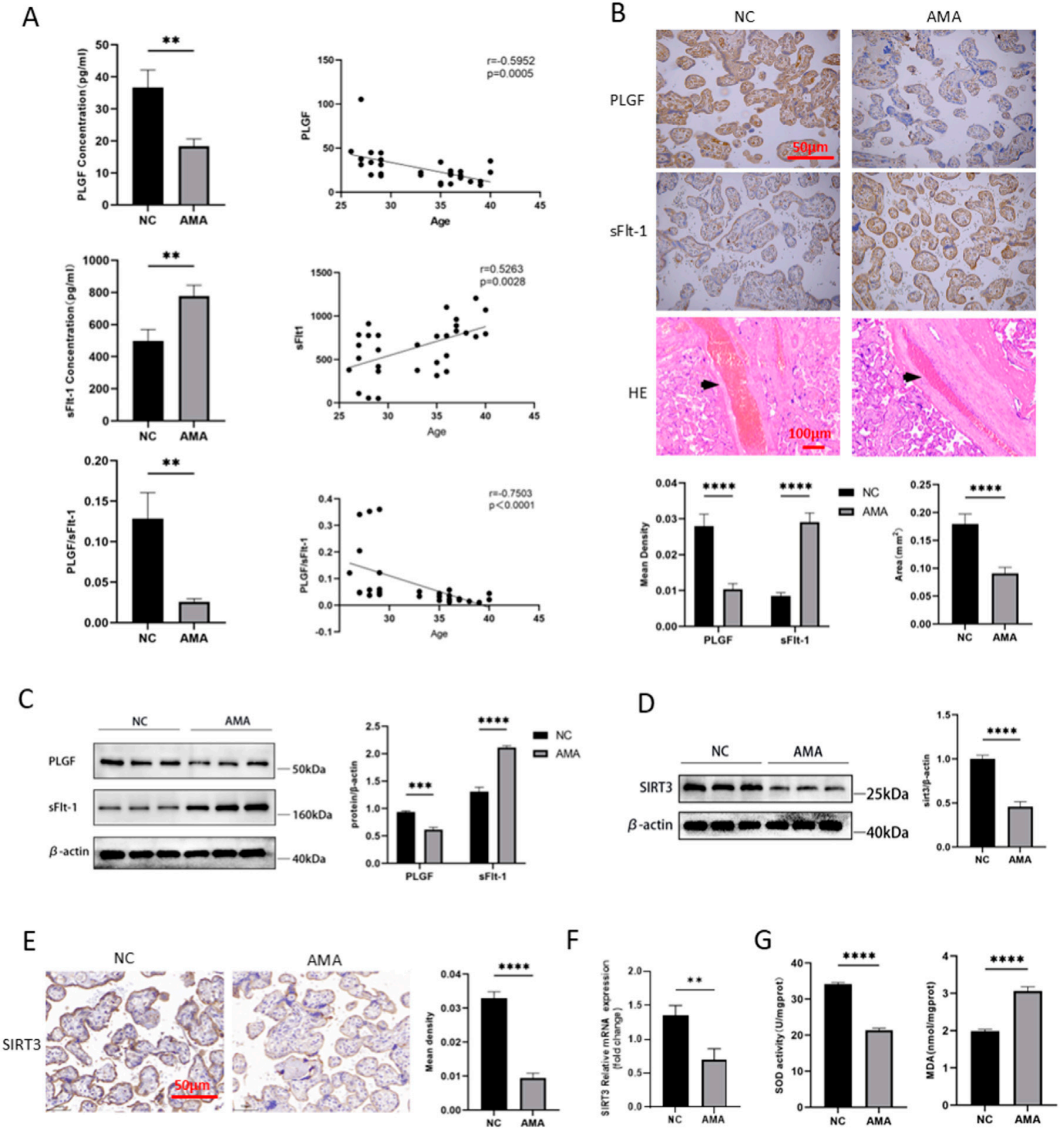
Verification of Placental Angiogenesis, Oxidative Stress, and SIRT3 Expression in AMA **(A)** Serum PLGF and sFlt-1 levels and their correlation with age in AMA and NC groups. **(B)** and **(C)** Placental PLGF, sFlt-1 levels and decidual vascular area in AMA and NC groups. **(D)** and **(E)** SIRT3 protein levels of placenta in AMA and NC groups. **(F)** SIRT3 mRNA of placenta in AMA and NC groups. **(G)** Superoxide dismutase level and lipid peroxidation level of placenta in AMA and NC groups.

The baseline characteristics of the included individuals used in the placental transcriptome sequencing and verification experiments are detailed in Supplementary Table S1. There were no statistically significant differences in the basic information of the patients between the two groups, except for the age. The serum ELISA results indicated significant differences in the levels of placental growth factor (PLGF) and soluble fms-like tyrosine kinase-1 (sFlt-1) between the AMA group (n = 15) and NC group (n = 15). The PLGF level in the high-risk pregnancy group was lower than that in the NC group (*p* = 0.005), as shown in Figure 5A. Conversely, the sFlt-1 level in the AMA group was significantly higher compared to the NC group. Additionally, the PLGF/sFlt-1 ratio of the AMA group was higher than that of the NC group (*p* = 0.000). Correlation analyses revealed a positive correlation was observed between age and serum sFlt-1 levels (r = 0.5263, *p* = 0.0028). Furthermore, the result demonstrated strong inverse correlations between maternal age and both serum PLGF levels (r = −0.5952, *p* = 0.0005) and the PLGF/sFlt-1 ratio (r = −0.7503, *p* < 0.0001).

As shown in Figure 5B, by Immunohistochemical staining, PLGF expression in the placenta was decreased while sFlt-1 expression was increased in the AMA group (n = 15) compared to the NC group (n = 15), significantly (*p* < 0.005). Furthermore, HE staining revealed a reduction in the decidual vascular space in the AMA group (n = 30) (*p* < 0.005). The expression levels of PLGF and sFlt-1, as determined by Western blot, showed the same trend as immunohistochemical staining (Figure 5C). These results indicate that angiogenesis is abnormally impaired in placentas of AMA, with concomitant dysregulated remodeling of spiral arteries.

In previous results, SIRT3 as the predominant hub gene, is involved in regulating oxidative stress response within mitochondrial function. Compared to the NC group (n = 6), both protein and mRNA levels of SIRT3 in the placenta of AMA pregnancies were significantly decreased (*p* < 0.05, Figures 5D–F). Furthermore, the levels of MDA, a marker of oxidative stress, were found to be elevated in the placentas of AMA pregnancies (*p* < 0.05, Figure 5G), while the activity of SOD, an important antioxidant enzyme, was significantly lower compared to the NC group (n = 30) (*p* < 0.05). SIRT3 downregulation in the placenta of AMA pregnancies suggests a potential mechanism of oxidative stress damage and poor placental perfusion. The decreased antioxidant capacity due to low SIRT3 levels may contribute to oxidative stress-related placental injuries, which are associated with adverse pregnancy outcomes in AMA pregnancies.

## 4 Discussion

MVM increases the risk of specific pregnancy complications. Therefore, we collected placental pathology results from 129 cases to identify risk factors associated with MVM development. Statistical analysis of pathological results demonstrated that AMA is an independent risk factor for inadequate maternal vascular perfusion in the placenta. This finding aligns with research conducted by Miremerg et al. although a consensus in the literature regarding the incidence of maternal MVM between AMA and NC groups has yet to be established (Torous and Roberts, 2020). Then, we performed differential expression analysis of genes in placentas from AMA and NC groups using high-throughput sequencing technology. A total of 731 statistically significant DEGs were identified in the AMA group compared to the NC group, with 445 genes showing upregulation and 286 genes downregulation. GO pathway analysis indicated that these DEGs were primarily enriched in processes related to energy metabolism, oxidative stress, angiogenesis, and NAD(P)H metabolism. Notably, the oxidative stress and angiogenesis pathways were highlighted as critical biological processes. We hypothesize that abnormalities in oxidative stress and angiogenesis may significantly impact placental vascular perfusion and placental function in pregnancies associated with AMA.

To further verify our hypothesis, we performed clustering analysis using gene expression data from the GSE133592 dataset, identifying six core genes: SIRT3, TLR6, AOX1, ARG1, CRYAB, and HGF. Among these, SIRT3, recognized as a classic antioxidant gene, exhibited the highest degree score within the network. Given that placentas from AMA pregnancies exhibit high levels of oxidative stress, this condition is theoretically linked to vascular development impairments within the placenta, potentially associated with abnormal SIRT3 expression. TLR6 forms a heterodimer with TLR2, and its overactivation may trigger inflammation via NLRP3 inflammasome induction (Kaneko et al., 2019; Unterberger et al., 2023), disrupting placental inflammatory balance and significantly increasing spontaneous preterm birth risk (Rasheed et al., 2020). Acting as a molecular chaperone, CRYAB counteracts oxidative stress-induced protein misfolding and participates in early placental development and trophoblast differentiation, potentially through stabilizing the HSP27-BAX complex (Mineva et al., 2008; Shochet et al., 2016). Preeclampsia (PE) patients exhibit significantly lower serum arginase-1 (Arg-1) levels than healthy controls. As Arg-1 is a key immunosuppressive effector of granulocytic myeloid-derived suppressor cells (G-MDSCs), its reduction directly correlates with impaired G-MDSC expansion in PE. This suggests Arg-1-mediated immunoregulatory dysfunction contributes to PE pathogenesis (Pinto-Souza et al., 2021; Wang et al., 2018). Hepatocyte growth factor (HGF), regulating placental angiogenesis and trophoblast invasion, shows aberrant expression closely linked to PE development (Li et al., 2022; Travaglino et al., 2019). While no study definitively links aldehyde oxidase 1 (AOX1) to gestational disorders, current evidence suggests AOX1 may help maintain normal placental structure and function by regulating redox balance and energy metabolism. However, the specific nature and strength of this association require further investigation.

Additionally, we observed a significant decrease in decidual vascular structures in the placentas of AMA pregnancies. Reduced decidual vascular area reflect insufficient remodeling of the uterine spiral arteries and MVM, which can lead to placental ischemia and oxidative damage due to accelerated flow rates in the intervillous space (Jung et al., 2022; Zur et al., 2020). Research exploring the connection between AMA and inadequate maternal vascular perfusion remains in its developmental stages, and factors such as regional variations, climate differences, dietary habits, and lifestyle choices may influence placental blood circulation. Beyond direct placental pathology, the serum PLGF/sFlt-1 ratio serves as a useful index for assessing placental angiogenesis (Dymara-Konopka et al., 2023). PLGF, primarily produced by trophoblast cells, plays a pivotal role in promoting placental angiogenesis, trophoblast proliferation, and invasion. Conversely, sFlt-1 inhibits the activity of vital angiogenic factors by binding to VEGF and PLGF, exacerbating endothelial damage and suppressing angiogenesis. Our findings indicated a concomitant downregulation of PLGF and an upregulation of sFlt-1 in the placental tissue and serum of AMA pregnancies, leading to a decreased PLGF/sFlt-1 ratio. This molecular-level evaluation suggests the presence of vascular development impairments in placentas associated with AMA. Significant accumulation of placental vascular damage can lead to functional deficiencies, thereby increasing the risk of pregnancy complications such as preeclampsia, fetal growth restriction, and stillbirth (Odame Anto et al., 2018).

SIRT3 is a major mitochondrial nicotinamide adenine dinucleotide (NAD^+^)-dependent deacetylase that positively regulates numerous cellular processes, including energy metabolism, mitochondrial biogenesis, and oxidative stress protection. Predominantly expressed in metabolically active organs such as the heart, kidneys, and liver (Zhang J. et al., 2020; Zhou et al., 2022). SIRT3 has been linked to lifespan extension and possesses protective roles against aging (Sack and Finkel, 2012; Zhao et al., 2020). Although the placenta is a transient yet highly metabolically active organ that facilitates maternal-fetal nutrient and energy exchange, the precise expression and function of SIRT3 within the placental tissue have yet to be fully elucidated. Existing studies indicate that SIRT3 expression is downregulated in preeclamptic placentas. This downregulation of SIRT3 increases necrosis and apoptosis in trophoblast cells while simultaneously impairing trophoblast invasion and migration (Yu et al., 2022). In placentas from fetuses with intrauterine growth restriction, the levels of the active form of SIRT3 protein are significantly reduced. This leads to impaired mitochondrial biogenesis and dysregulation of mitochondrial homeostasis, which in turn affects placental function (Naha et al., 2020). The aberrant expression of SIRT3 in placentas affected by pregnancy complications such as preeclampsia exerts well-documented detrimental effects. Therefore, SIRT3 may serve as a potential target for both the diagnosis and treatment of preeclampsia. This research, grounded in sequencing results, further confirms that SIRT3 is predominantly localized in placental tissue. Notably, SIRT3 is significantly downregulated in placentas of AMA pregnancies, which may be linked to heightened levels of MDA and reduced SOD activity, indicative of increased oxidative stress. This state of oxidative stress may be closely associated with the abnormalities observed in placental vascular development in groups with AMA.

This study has several limitations. Firstly, the sample size for sequencing was relatively small, with only six placental tissue samples subjected to high-throughput sequencing. This limitation may affect the generalizability of our findings. Secondly, our research comprises only preliminary clinical correlation analyses, and further studies are warranted to delve deeper into the mechanisms by which SIRT3 mediates oxidative stress damage and contributes to inadequate vascular perfusion in the placenta. Despite these limitations, our research represents the first exploration of the association between SIRT3 expression levels and oxidative stress damage in the placenta, as well as adverse pregnancy outcomes in AMA. This preliminary evidence lays a critical theoretical foundation for considering SIRT3 as a risk marker for conditions associated with AMA, and identifies it as a potential target for intervention strategies aimed at improving maternal and fetal health outcomes. Future investigations will focus on elucidating the pathways through which SIRT3 impacts placental health, thereby enhancing our understanding of its role in pregnancy complications associated with AMA.

Although our study revealed molecular abnormalities in placental angiogenesis, energy metabolism, and oxidative stress in advanced maternal age (AMA) pregnancies, and identified reduced SIRT3 expression as a potential mechanism, several limitations remain. While WGCNA identified multiple AMA-associated co-expression modules and we functionally validated SIRT3, other key genes or regulatory networks within these modules require further investigation. Then, we only observed decreased SIRT3 expression and increased oxidative stress at the tissue level; functional studies using cellular or animal models are still needed to directly demonstrate the role of SIRT3 in placental vascular dysfunction. Addressing these limitations will be a focus of our future research.

## 5 Conclusion

In this study, we conducted high-throughput sequencing analysis of placental tissues from AMA and NC pregnancies. Our findings indicated significant differences in oxidative stress and angiogenesis-related pathways between the two groups, and identified key differential genes such as SIRT3. The observed downregulation of SIRT3 expression in placentas from AMA pregnancies may be associated with oxidative stress imbalances and impaired angiogenesis; however, further studies are needed to clarify the underlying mechanisms and causal relationships. Considering the increased risk of adverse pregnancy outcomes in AMA, our results provide preliminary insights into possible pathological processes at play. These findings may offer perspectives for future research on surveillance and intervention strategies in managing pregnancies at advanced maternal age.

## Data Availability

The datasets presented in this study can be found in online repositories. The names of the repository/repositories and accession number(s) can be found in the article/Supplementary Material.
