# Supplementary material for: High-throughput sequencing unravels placental vascular dysfunction and oxidative stress as mechanistic drivers of advanced maternal age-associated pregnancy
Source: Front Genet. 2025 Aug 13;16:1636834. doi: 10.3389/fgene.2025.1636834 (PMC12381460; doi:10.3389/fgene.2025.1636834)
Supplement: Supplementary file 1 [file Table1.docx]

**Supplementary Table 1**: Comparison of General Information of Patients in Different Groups

| Characteristics | NC（n=20） | AMA（n=20） | P Value |
| --- | --- | --- | --- |
| Age | 27.50±1.76 | 36.70±1.81 | <0.0001^z^ |
| Primigravida |  |  | 0.751^k^ |
| Yes | 14 | 12 |  |
| No | 10 | 12 |  |
| Primipara |  |  | 0.407^k^ |
| Yes | 18 | 15 |  |
| No | 2 | 5 |  |
| Systolic pressure (mmHg) | 117.6±11.20 | 119.6±12.62 | 0.590^t^ |
| Diastolic pressure (mmHg) | 73.35±11.10 | 75.45±75.45 | 0.483^t^ |
| BMI (kg/m^2^) | 27.70±4.65 | 27.16±3.306 | 0.673^t^ |
| Pre - pregnancy weight (kg) | 60.83±11.06 | 57.10±10.19 | 0.154^z^ |
| Height (cm) | 164.1±5.11 | 162.8±4.538 | 0.547^z^ |
| newborn weight (g) | 3365±413.89 | 3291±368.16 | 0.556^t^ |
| Apgar score (1min) | 8.900±0.31 | 9.0±0.00 | 0.487^z^ |
| Apgar score (5mins) | 9.700±0.47 | 9.600±0.50 | 0.741^z^ |

Note: A P-value less than 0.05 indicates that the difference is statistically significant. "t" represents the application of the t-test, "z" represents the application of the rank sum test, and "k" represents the application of the chi-square test.
